# Identification of pathogenic mutations in 6 Chinese families with multiple exostoses by whole-exome sequencing and multiplex ligation-dependent probe amplification: Case series

**DOI:** 10.1097/MD.0000000000015692

**Published:** 2019-05-17

**Authors:** Xigui Long, Zhuo Li, Yanru Huang, Li Zhang, Weigang Lv, Yanling Teng, Siyuan Linpeng, Desheng Liang, Lingqian Wu

**Affiliations:** a Center for Medical Genetics & Hunan Key Laboratory of Medical Genetics, School of Life Sciences, Central South University; b Hunan Jiahui Genetics Hospital, Changsha, Hunan, PR China.

**Keywords:** *EXT1*, *EXT2*, hereditary multiple exostoses, MLPA, whole-exome sequencing

## Abstract

**Rationale:**

Hereditary multiple exostoses (HMEs) is an autosomal dominant skeletal disorder.

**Patient concerns:**

Six probands of the 6 unrelated Han Chinese families were identified as having HME. These patients had exostoses at multiple sites and significantly affected joints malformation and movement.

**Diagnoses:**

Hereditary multiple exostoses.

**Interventions:**

To detect the genetic mechanism of HME in 6 unrelated Chinese families, whole-exome sequencing (WES) and multiplex ligation-dependent probe amplification (MLPA) were used after genomic DNA was isolated from peripheral blood leucocytes. Point mutations identified by these methods were verified by Sanger sequencing after PCR amplification.

**Outcomes:**

Six mutations in the *EXT1* and *EXT2* genes were identified, including a heterozygous deletion mutation from exon 2 to exon 8 (Family 1), a c.448C>T, p.(Gln150X) heterozygous nonsense mutation (Family 4), a c.1057-2A>T heterozygous splicing substitution (Family 5), and a c.1468dupC, p.(Leu490fs519X) (Family 6) heterozygous duplication mutation in the *EXT1* gene in addition to a heterozygous deletion mutation from exon 2 to exon 3 (Family 2) and a c.1197C>G, p.(Tyr399X) heterozygous nonsense mutation (Family 3) in the *EXT2* gene.

**Lessons:**

Overall, we identified 5 novel mutations and 1 recurrent mutation in the *EXT1* and *EXT2* genes in 6 Chinese families with HME. Our findings expand the mutational spectrum of the *EXT1* and *EXT2* genes and are useful for genetic counseling and prenatal diagnosis.

## Abstract 2

Supplemental Digital Content is available in the text

## 1 Introduction

Hereditary multiple exostoses (HMEs) (MIM #133700, #133701, and %600209), also known as multiple osteochondromas (MOs), is characterized by the presence of multiple exostoses that mainly exist in the metaphysis of long bones. These regions include the humerus, proximal tibia and fibula, and distal femur, with occurrence rates ranging from approximately 70% to 90%.^[1,2]^ These deformities, such as unequal limb length and form, forearm malformation, valgus malformation of the legs and scoliosis, are the main clinical symptoms of patients.^[3,4]^ The penetrance of HME is estimated to be approximately 100%, and the incidence is estimated to be approximately 1 to 2/100,000.^[2]^

HME patients may present mild symptoms or severe symptoms, such as pain and limited mobility, and some patients must undergo multiple surgeries.^[5]^ A mutation in *EXT1* (MIM #608177), *EXT2* (MIM #608210), or exostosin-3 (*EXT3*) genes is the cause of HME. The EXT1 and EXT2 are homologous and share an approximately 70% amino acid identity with each other.^[6,7]^ They encode heparin sulfate glycosyl transferases that are composed of 746 and 718 amino acids, respectively,^[7–10]^ and play an important role in heparan sulfate (HS) synthesis. The synthetic complex is an essential factor in the signal transduction cascade for chondrocyte cell differentiation, ossification, and apoptosis.^[11,12]^ Approximately 70% to 95% of affected individuals have a pathogenic mutation in the *EXT1* or *EXT2* gene.^[13]^ To date, hundreds of mutations in the *EXT1* and *EXT2* genes related to HME have been reported.

HME has high heritability and teratogenicity. It is necessary to perform genetic diagnosis or prenatal diagnosis, as the offspring of patients have a 50% higher risk of developing the disease. Due to the lack of mutation hotspots and large size of the *EXT1* and *EXT2* genes, WES and MLPA were performed in this study.

## 2 Patients and methods

### 2.1 Patients

**Figure 1:**
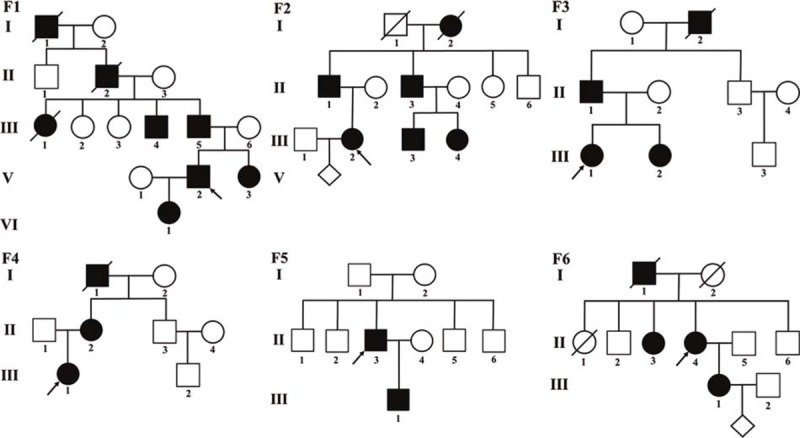
Pedigree of 6 Chinese families with HME. Circles and squares indicate females and males, respectively. Empty and filled symbols indicate unaffected individuals and affected individuals, respectively. Arrows and diagonal lines indicate the probands and deceased individuals, respectively. Rhombuses indicate fetuses.

Six probands of the 6 unrelated Han Chinese families were identified as having HME (Fig. 1). Family histories were collected. Related clinical examinations, such as X-ray imaging, and physical examinations were carried out. For the diagnosis of HME according to the current criteria,^[14]^ at least 2 exostoses must be detected by radiography or palpation in the metaphysis of long bones. Thirty-nine blood samples from these families were obtained, which included 21 affected individuals and 18 unaffected individuals. Two hundred unrelated normal individuals were recruited as normal controls. This study was consistent with the tenets of the Declaration of Helsinki and was approved by the ethics committee of the Hunan Jiahui Genetics Hospital (the ethical approval number: 2017011502). Informed consent was obtained from the patient for publication of this case report and accompanying images.

### 2.2 Exome capture and sequencing

Genomic DNA was extracted from peripheral blood cells by the phenol-chloroform extraction method. Exon-containing fragments were enriched with the xGen Probes Panel (Integrated DNA Technologies, Coral, IL) and sequenced with the HiSeq 2000 platform (Illumina, San Diego, CA) by the paired-end method. A mean exome coverage of more than 100x was obtained. The sequencing depath was greater than 10x for ninety seven percent of capture regions. Data analysis was performed using the VeritaTrekker and Enliven Bioinformatics analysis pipeline. Variants identified were filtered through the process (see Supplement-Fig. 1). Splicing mutations were checked using Human splicing finder 3.0 (http://umd.be/HSF3/).

### 2.3 Sanger sequencing

Four pair primers (see Supplement-Table 1) were designed for standard PCR assays using Primer PREMIER Version 5.0 (Premier Biosoft International, Palo Alto, CA). The amplified DNA fragments were directly sequenced by Sanger sequencing (Biosune, http://www.biosune.com). DNASTAR software (Madison, WI) was used to analyze the sequencing results by comparison with the reference sequences.

### 2.4 Multiple ligation-dependent probe amplification (MLPA)

The commercial kit #P215-B3 (MRC-Holland, Amsterdam, the Netherlands) was used following the manufacturer's instructions. An ABI 3130 sequencer (Applied Biosystems, Foster City, CA) was used for capillary electrophoresis. Coffalyser.Net software was used for the analysis of MLPA data. The proportion of each peak height was calculated. The normal copy number range (dosage quotient) is between 0.8 and 1.2; a ratio from 0.4 to 0.65 indicates heterozygous deletion, and a ratio from 1.3 to 1.65 indicates heterozygous duplication. Each positive result was repeated.

### 2.5 Bioinformatics analysis

The structures of genes and proteins were analyzed by UniProtKB (http://www.uniprot.org/) and UCSC (http://genome.ucsc.edu/). The physical and chemical parameters of proteins were analyzed by ProtParam (http://web.expasy.org/protparam/).

## 3 Results

### 3.1 Description of patients

**Figure 2:**
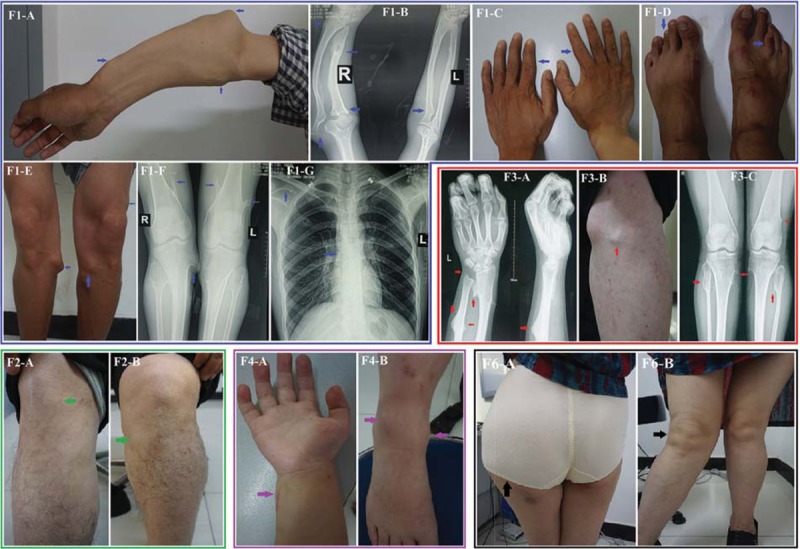
Appearance and radiology results of the probands. F1: (A–G) Obvious right forearm deformities (protruding from the upper lateral radius, forearms bend inward), left ulnar tuberosity bulges (F1-A), a relatively truncated and curved ulna and radius (F1-B), short metacarpals (F1-C), third brachymetatarsia (F1-D), multiple exostoses around the knees (F1-E), localized bony protrusions on the distal femur, proximal tibia and fibula (F1-F), mild symptoms of costal and scapular exostoses and scoliosis (F1-G) were observed in the proband (V: 2) and are indicated with blue arrows. F2: (A, B) Exostoses that occurred around both knees (F2-A, F2-B) were found in the proband (III:2) and are indicated with green arrows. These exostoses had been partly surgically removed. F3: (A–C) X-rays showed multiple bony protrusions in the left distal radius and ulna (F3-A), as well as exostoses around both knees (F3-B). X-rays showed localized bony protrusions around both knee joints and slightly narrowed joint spaces (F3-C) (proband: III:1, indicated with red arrows). F4: (A, B) Exostoses at multiple sites, including the right bottom of the radius and wrist (F4-A) and the right side of the lower tibia (F4-B) (proband: III:1, indicated with purple arrows). F6: (A, B) Exostoses that were located on the upper side of the left femur (F6-A), the coxa valga and mild scoliosis were observed in the proband (II: 4, F6) (F6-B) (proband: II: 4, indicated with black arrows).

In family 1 (F1) (Fig. 1-F1), the proband (V: 2) was a 28-year-old male with a height of 169 cm from Anhui province. Exostoses began to appear around both knees from the age of 5. He exhibited an obvious Madelung-like forearm deformity of the right forearm (protuberances in the upper lateral radius, with forearms bent inward), left ulnar tuberosity bulges, bilateral genu valgum deformities, multiple exostosis around the knees bilaterally, bilateral short metacarpals, and third brachymetatarsia. In addition, the left shoulder blade was higher than the right shoulder blade, and X-rays showed relatively truncated and curved ulnas and radii, mild scoliosis, and localized bony protrusions on the distal femurs, proximal tibias and fibulas, right scapula and ribs (Fig. 2: F1A–G). The other family members, I:1 (deceased), II:2 (deceased), III: 1 (deceased), III: 4, III: 5, V: 3, and VI:1, exhibited similar manifestation.

In family 2 (F2) (Fig. 1-F2), the 27-year-old female proband (III:2), who came from Hunan province, had a height of 156 cm. Progressive exostoses occurred around both knees, wrist joints, and forearms and presented early in her childhood. These exostoses had been surgically removed (Fig. 2: F2A, B). Excisional biopsy was benign by pathological detection. Her younger cousins (III: 3, III: 4), father (II:1), uncle (II:3), and grandmother (I:2, deceased) exhibited similar manifestations.

In family 3 (F3) (Fig. 1-F3), the proband (III:1), with a height of 160 cm, was a 30-year-old female from Shanxi province. Exostoses occurred around both knees, the right scapula, distal radius, and ulnas, and the right side of the bottom tibia had been progressively worsening since the age of 6. X-rays showed localized bony protrusions around both knee joints, slightly narrowed joint spaces, and multiple bony protrusions in the left distal radius and ulnar (Fig. 2: F3A–C). She had undergone multiple operations. Her father (II:1), sister (III:2), and grandfather (I:2, deceased) had similar clinical manifestations.

In family 4 (F4) (Fig. 1-F4), the proband (III:1) was a 6-year-old female from Jiangxi who complained of walking with a valgus gait since the age of 2 years. Exostoses at multiple sites were present, including the right side of the lower tibia and the right bottom of the radial and wrist joint (Fig. 2: F4A, B). The proband's mother (II:2) and grandfather (I:1, deceased) also exhibited exostoses at multiple sites.

In family 5 (F5) (Fig. 1-F5), the proband (II:3) was a 43-year-old male with a height of 158 cm from Guangxi province. He had discovered exostoses at 3 years of age. He had exostoses at multiple sites and obvious right forearm malformation. The left femoral neck, bilateral femoral heads, bilateral upper end of the tibias, and fibulas had multiple bony protrusions. The pathological examination indicated osteochondroma after surgical resection. His son (III:1) also had exostoses, mostly around the knee joints, with no apparent pain, and they became increasingly obvious during adolescence.

In family 6 (F6) (Fig. 1-F6), the proband (II:4) presented to our clinic with the complaint of pain caused by exostoses around the proximal joints of the extremities since the age of 9. She was 55 years old, had a height of 156 cm, and was from Sichuan province. The abnormal gait had become worse with aging. The exostoses in the upper side of the left femur were obviously increased. The coxa valga was obvious (Fig. 2: F6A, B). Her daughter (III:1) had similar clinical features. Exostoses appeared at the distal end of the right radius at 4 years of age and obviously increased. The proximal tibias were also affected, and she also had slight pain and an abnormal gait. When the proband came to our clinic, the patient (III:1) was 7 weeks pregnant, and a genetic test and subsequent prenatal diagnosis were performed.

### 3.2 Identification of these mutations

**Figure 3:**
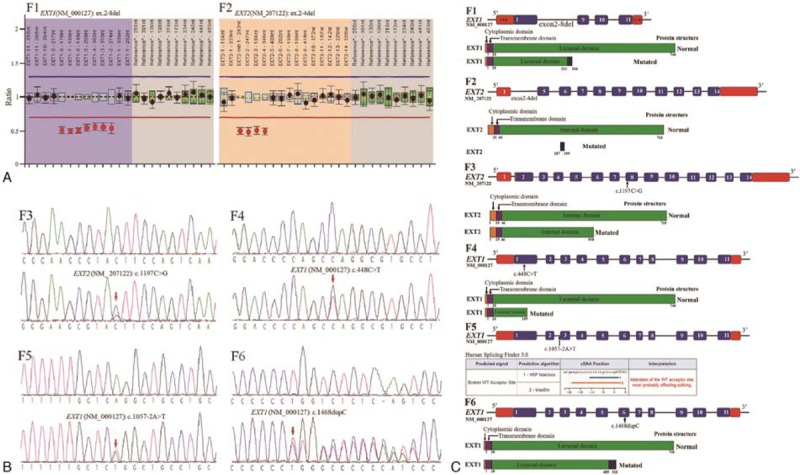
Mutation spectrum of the *EXT1/EXT2* genes and pathogenicity analysis of these mutations. (A) A heterozygous deletion of the *EXT1* gene from exon 2 to exon 8 was detected in F1, and a heterozygous deletion of the *EXT2* gene from exon 2 to exon 4 was detected in F2. (B) A c.1197C>G heterozygous nonsense mutation in the *EXT2* gene in F3, a c.448C>T heterozygous nonsense mutation in *EXT1* in F4, a c.1057-2A>T heterozygous splicing substitution in F5, and a c.1468dupC heterozygous duplication mutation in *EXT1* in F6 were detected in affected individuals of the 6 Chinese families with HME, respectively. The mutations detected in the probands were not detected in the unaffected individuals or the 200 unrelated normal individuals. (C) Schematic drawing of the normal and mutated region of the *EXT1* and *EXT2* genes and proteins (based on the description in UniProtKB-Q93063, http://www.uniprot.org/uniprot/Q93063). Exons are marked in blue (UTRs are marked in red). Orange, purple, and green indicate the cytoplasmic domain, transmembrane domain, and luminal domain, respectively. Prediction for c.1057-2A>T in the *EXT1* gene by Human Slicing Finder 3.0 (C-F5).

In this study, 6 mutations in *EXT1* and *EXT2* were detected in 6 unrelated Chinese families with HME and are summarized in detail in Table 1. In F1and F2, no possible pathogenetic variation was detected by WES. MLPA was then used, resulting in the identification of a heterozygous deletion of the *EXT1* gene from exon 2 to exon 8 and a heterozygous deletion of the *EXT2* gene from exon 2 to exon 4 in the affected individuals of these 2 families, respectively (Fig. 3-F1, F2). In F3, a c.1197C>G, p. (Tyr399X) heterozygous nonsense mutation in exon 8 of *EXT2* was identified in the patients (Fig. 3-F3). In F4, a c.448C>T, p. (Gln150X) transition was observed in exon 1 of the *EXT1* gene in the patients (Fig. 3-F4). In F5, a c.1057-2A>T splicing mutation was detected in the exon 3/intron 3 boundary of the *EXT1* gene in the proband (II:3) (Fig. 3-F5). In F6, a recurrent small duplication mutation was identified in exon 6 of the *EXT1* gene and defined as c.1468dupC (Fig. 3-F6). The prenatal diagnosis was performed after amniocentesis at the 18th week. The same heterozygous duplication mutation was detected in the amniocytes, and the pregnant woman (III:1, F6) terminated the pregnancy. The mutations described above were verified by verification of pedigree. Compared with the affected individuals, the unaffected individuals and 200 unrelated normal individuals did not have the same mutation.

**Table 1:**
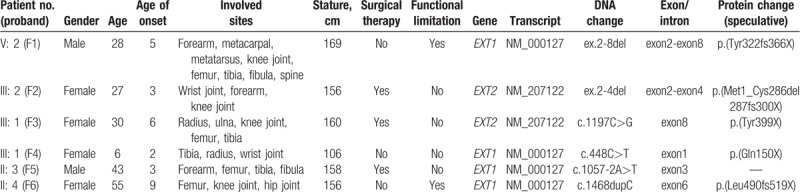
Clinical data and mutations identified in *EXT1* and *EXT2* in 6 Chinese probands with hereditary multiple exostoses.

### 3.3 Bioinformatics analysis of mutants

On the basis of the description of UniProtKB-Q16394/Q93063 (http://www.uniprot.org/uniprot/), schematic diagrams of the mutant proteins were drawn, showing the normal and mutated regions of EXT1 and EXT2 (Fig. 3C). These speculative mutants, such as p. (Gln150X) of EXT1 and p. (Met1_Cys286del287fs300X), p. (Tyr399X) of EXT2, had higher instability indices, and the aliphatic index and average hydropathicity of p.(Tyr322fs366X), p.(Gln150X), and p.(Leu490fs519X) in EXT1 were significantly reduced (supplement-Table 2).

## 4 Discussion

HME is a heterogenic monogenic disorder with variable numbers and sizes of exostoses and locations of diseased bones. *EXT1* and *EXT2* mutations have been detected in approximately 70% to 95% of patients with HME, with *EXT1* accounting for 56% to 78% and *EXT2* accounting for 21% to 44% of cases.^[13]^ To date (May 5, 2018), in the Multiple Osteochondroma Mutation Database (http://medgen.ua.ac.be/LOVDv.2.0/home.php), the numbers of variations in *EXT1* and *EXT2* are 436 and 223, respectively. Most of these mutations are detrimental and are predicted to produce truncated proteins. As shown in Fig. 3C, the normal EXT protein is composed of 3 domains, namely, the cytoplasmic domain, transmembrane domain, and luminal domain. In our study, WES was performed first, and when no mutation was found, MLPA was performed to detect the presence of copy variation in the *EXT1* and *EXT2* genes. In this study, 6 mutations were detected in the *EXT1*and *EXT2* genes, 5 of which are novel mutations. The heterozygous deletion from exon 2 to exon 8 in the *EXT1* gene in F1 may result in frameshift mutation from the 322nd amino acid residue and stop gain at 366th, which may result in loss of most of the EXT1 luminal domain. The heterozygous deletion from exon 2 to exon 4 in the *EXT2* gene in F2 is speculated to result in the loss of almost entire amino acids. Both of them are large-fragment exon deletions that inevitably change the normal protein structures. The c.448C>T nonsense mutations that were detected in F4 may lead to a truncated protein at the 150th amino acid in EXT1. The c.1197C>G nonsense mutations detected in F3 are similar to those at the site of c.1197C>A, which was reported to be associated with HME in 2009.^[13]^ Both the c.1197C>G and c.1197C>A mutations can change the amino acid codon to a termination codon at the 399th amino acid in EXT2. The C-terminus of the EXT proteins may be the catalytic regions of the enzyme,^[14]^ and nonsense-mediated mRNA decay may also cause haploinsufficiency. These 2 nonsense mutations identified here are speculated to result in the loss of these important catalytic regions and, inevitably, the loss of some or all of the function of these regions. Splice site mutations can induce exon skipping, influence mRNA splicing, or activate cryptic splice sites. Currently, 51 *EXT1* splice site mutations have been reported in the Human Gene Mutation Database (HGMD). In our study, a novel c.1057-2A>T heterozygous splicing mutation was detected in the exon 3/intron 3 boundary of the *EXT1* gene in F5. These c.1057-2A>C^[15]^ and c.1057-2A>G^[16]^ splicing substitutions that are associated with HME were reported, which indicated the position of c.1057-2 as a key splicing site. We used Human Splicing Finder 3.0 to analyze the splicing mutation. The predicted results showed that the mutation would alter the wild-type (WT) acceptor site and likely affect splicing. Perhaps due to the difficulty of collecting fresh specimens from patients, only a few mutations in *EXT1* and *EXT2* have been studied at the RNA level.^[17,18]^ The c.1468dupC recurrent frameshift mutation in the *EXT1* gene was reported in 2001^[19]^ and was also identified in F6 in our study. The mutation may cause the replacement of 30 amino acid residues and further produce truncated proteins that truncated at the 519th amino acid residue. These changes in the physical and chemical parameters of mutants, such as the higher instability index, lower aliphatic index, and hydropathicity, indicate the decreased stability and increased degradability of the mutants.

There is a high rate of knee deformity in patients with HME, with approximately one-third of patients developing genu valgum.^[20]^ Bowing deformities of the forearms are even more frequent and are observed in 30% to 60% of patients with HME.^[2,21,22]^ Other skeletal deformities include limb length discrepancy, valgus deformity of the lower extremities, and scoliosis. Obvious forearm deformities and multiple exostoses around the knees were also observed in these probands (V: 2, F1 and III:1, F3). Conspicuous short metacarpals, third brachymetatarsia, and scoliosis were observed in the proband (V: 2, F1). Obvious coxa valga and mild scoliosis were observed in the proband (II: 4, F6). HME can lead to a disproportionate short stature due to the influence of long bone growth plates. We also observed the symptoms of these probands, 4 of whom (III:2, F2; III:1, F3; II: 3, F5; II: 4, F6) had a height of less than 160 cm. Costal exostoses are mostly asymptomatic, but they can rarely cause intrathoracic complications, including hemothorax and pneumothorax. We did not observe such complications in our patients, except for a patient (V: 2, F1) who had mild symptoms of costal and scapula exostoses. Due to the small sample size, we did not determine the relationship between the genotype and phenotype. However, it is possible that not only the genetic factors but also the living conditions and social environments all contribute to the phenotypic heterogeneity. These mutations in the *EXT1* and *EXT2* genes may destroy the encoding area structure, impact RNA transcription levels, and subsequently lead to haploinsufficiency.^[13,23,24]^ The encoded mutations may influence hetero-oligomeric complex formation and weaken catalysis in the polymerization of HS. This complex is an essential factor in the signal transduction cascade for regulation of chondrocyte differentiation, ossification, and apoptosis.^[12,14]^ Mutations in the *EXT1* and *EXT2* genes may lead to structural changes in EXT glycosyltransferases and, subsequently, disturb interactions, lower enzymatic activity and the production of HS, disturb EXT1/EXT2 complex formation, and lead to the occurrence of HME.^[13,25]^ These detected variants in the 6 unrelated Chinese families were not found in ExAC, 1000G, or ESP6500. They also cosegregated with the disease phenotype in these families and were assessed as pathogenic or likely pathogenic variants, according to the standards and guidelines for the interpretation of sequence variants of the American College of Medical Genetics and Genomics (ACMG), which suggests that they may be the genetic pathogenesis of HME.

Due to the heterogeneity of HME, as well as lack of mutation hotspots and the large size of the *EXT1*/*EXT2* genes, we effectively applied WES in these patients from 6 Chinese families with HME. Pathogenic mutations were detected in 4 of the patients, including all the point mutations that WES can assess. This could be a routine technique in molecular diagnosis for monogenic disorders when a patient's symptoms are atypical and heterogeneous in the near future.

In conclusion, we identified and evaluated 6 mutations in the *EXT1* and *EXT2* genes in 6 Chinese families with HME. Among them, 4 and 2 mutations were identified in *EXT1* and *EXT2,* respectively, 5 of which are reported here for the first time. HME exhibits high heritability and teratogenicity; we successfully performed prenatal diagnosis for the pregnant women and prevented the fetuses that may have suffered HME after birth from being born. This study indicates that WES is effective in diagnosing monogenic disorders, such as HME, and extends the mutational spectrums in the *EXT1* and *EXT2* genes in Chinese patients, which is advantageous for genetic counseling and subsequent prenatal diagnosis.

## Acknowledgment

We thank all the healthy individuals and the family members for their participation and support in this study.

## Author contributions

**Conceptualization:** xigui long.

**Data curation:** xigui long, Yanru Huang.

**Formal analysis:** xigui long, Zhuo Li.

**Funding acquisition:** Desheng Liang, Lingqian Wu.

**Investigation:** xigui long.

**Methodology:** xigui long.

**Project administration:** Weigang Lv, Desheng Liang, Lingqian Wu.

**Resources:** xigui long, Siyuan Linpeng.

**Software:** xigui long, Yanru Huang, Li Zhang.

**Supervision:** Lingqian Wu.

**Validation:** xigui long, Yanling Teng.

**Visualization:** xigui long.

**Writing – original draft:** xigui long.

**Writing – review & editing:** Zhuo Li, Desheng Liang, Lingqian Wu.

## Supplementary Material

Supplemental Digital Content
