# Supplementary material for: Identification of pathogenic mutations in 6 Chinese families with multiple exostoses by whole-exome sequencing and multiplex ligation-dependent probe amplification: Case series
Source: Medicine (Baltimore). 2019 May 17;98(20):e15692. doi: 10.1097/MD.0000000000015692 (PMC6531242; doi:10.1097/MD.0000000000015692)
Supplement: Supplemental Digital Content [file medi-98-e15692-s001.doc]

**Supplementary Information**

Supplement-Figure 1. Analysis process of whole exome sequencing data.


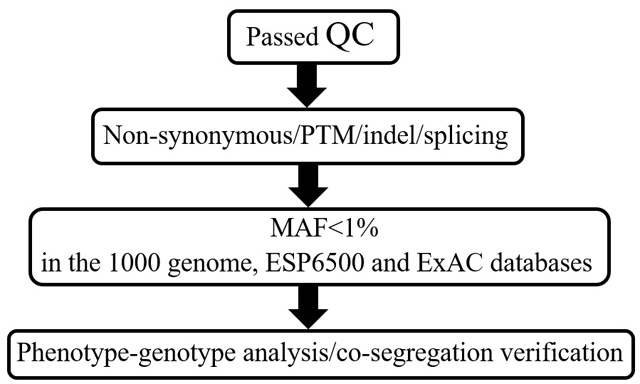


QC: Quality Control; PTM: Protein Function affected Mutation; MAF: Minor Allele Frequency.

Supplement-Table 1 The primers for Sanger sequencing

| **Family** | **Primers** | **Sequence** |
| --- | --- | --- |
| **F3** | *EXT2*-Forward | 5'-GCACCCCCATCCCTACAACTTT-3′ |
|  | *EXT2*-Reverse | 5'-GGAGGGTCATTCCATTCTTCATAGG-3′ |
| **F4** | *EXT1*-Forward | 5'-TGTCTCGCCCTTTTGTTTTATT-3′ |
|  | *EXT1*-Reverse | 5'-TGGTGTCTGATCCTATCCCTGT-3′ |
| **F5** | *EXT1*-Forward | 5'-AGAAATGGGGTTTTAGCATTCTAGG-3′ |
|  | *EXT1*-Reverse | 5'-AAAGTTTGGACGGGGGCAG-3′ |
| **F6** | *EXT1*-Forward | 5'-ATGAAGGGTGGTGAGTGTTGTGTG-3′ |
|  | *EXT1*-Reverse | 5′-GAATGAAAGGGAGTAGCAGGGTATG-3′ |

Supplement-Table 2 The physical and chemical parameters of WT and Mutants were analyzed by ProtParam

|  | **EXT1** | | | | **EXT2** | | |
| --- | --- | --- | --- | --- | --- | --- | --- |
| **Protein characteristics** | **WT** | **p.(Tyr322**  **fs366X)** | **p.(Gln150X)** | **p.(Leu490**  **fs519X)** | **WT** | **p.(Met1_Cys286del**  **287fs300X)** | **p.(Tyr399X)** |
| **Number of amino acids** | 746 | 366 | 149 | 519 | 718 | 12 | 398 |
| **Molecular weight** | 86254.75 | 41895.28 | 17269.55 | 59544.89 | 82254.53 | 1385.68 | 45297.02 |
| **Theoretical PI** | 9.16 | 9.4 | 9.46 | 9.05 | 6.12 | 7.86 | 6.71 |
| **Instablity index** | 52.26 | 48.89 | 67.12 | 51.35 | 45.29 | 130.08 | 49.41 |
| **Aliphatic index** | 81.03 | 65.03 | 58.32 | 79.65 | 90.91 | 32.5 | 92.51 |
| **Grand average of hydropathicity** | -0.371 | -0.674 | -0.726 | -0.41 | -0.202 | -0.242 | -0.182 |
